# Integrating positive psychology and spirituality in the context of climate change

**DOI:** 10.3389/fpsyg.2022.970362

**Published:** 2022-09-09

**Authors:** Christian R. Bellehumeur, Cynthia Bilodeau, Christopher Kam

**Affiliations:** School of Counselling, Psychotherapy and Spirituality, Faculty of Human Sciences, Saint Paul University, Ottawa, ON, Canada

**Keywords:** positive psychology, spirituality, climate change, wave metaphor, paradox, nature, eco-spirituality

## Abstract

In the context of climate change and its accompanying impact on stress and mental health, we argue that positive psychology (PP) may benefit from an integration of spirituality to better support people’s wellbeing. Starting with an overview of climate change’s impact on wellbeing and health, we explore the paradoxical and complex relationship between humans and nature. Following which, we will briefly define spirituality and present an evocative metaphor of the wave to portray the evolution of the field of PP. In our conclusive remarks, we argue that the field of PP has gradually become more open to integrate spirituality (since the first wave), as it evolves towards greater complexity (in its third wave). In addition to meaning, some spiritual perspectives potentially relevant to positive psychology facilitate an ecocentric view (i.e., eco-spiritualities) which allow for a better understanding of the paradoxical human-nature relationship, as we struggle to deal with the complex issues related to climate change.

## Introduction

“*We are the Earth. (…) When you realize there is no separation [between us, humans, and Earth], you fall completely in love with this beautiful planet.*”

– Thich Nhat Hanh, *Love letter to the Earth*

If the coronavirus pandemic has exposed the vulnerabilities of an interconnected and globalized world, its repercussions accentuate another more dreadful and permanent crisis that threatens our global community: climate change (Andrews and Hoggett, 2019). Despite the evidence, years of international negotiations and growing public concern (Bourgon, 2020), progress in reducing greenhouse gases is hardly palpable (IPBES, 2019; IPCC, 2022). Extreme weather events (i.e., heat waves and wildfires, droughts and floods, tornadoes and hurricanes) are increasing in frequency and intensity worldwide (Cianconi et al., 2020), while scientific indicators confirm the gradual deterioration of the planet (e.g., global warming, biodiversity loss, etc.) (Rockström et al., 2009a; IPCC, 2022). This burgeoning environmental crisis challenges our current ways of relating to the world (Nicolescu, 2014; Bourg, 2018). It beckons us to stop separating humans from the rest of nature (i.e., non-human forms of life) and rather to learn to integrate our own lives within the entire community of life on which our own future depends (Næss, 2008).

Adapting to the reality of climate change is a dynamic process which requires taking care of ourselves (i.e., our wellbeing) as well as harnessing our psycho-spiritual resources in order to adopt adequate pro-environmental behaviors. We are then left with the question: How do we foster wellbeing for all people while equally considering the health of the planet? In this article, we present a well-known Positive Psychology (PP) approach to fostering wellbeing in our current context and highlight the paradoxical relationship of humans with nature. As spirituality can often facilitate meaning making, it can also assist us in making sense of this paradox. We, therefore, argue the need to integrate spirituality within the broad PP framework to help us address and navigate the complex mental health issues arising from climate change.

## Climate change’s impacts on wellbeing and mental health

The consequences of climate change on people’s wellbeing continue to be substantial (Bélanger et al., 2019). Extreme weather events related to global warming have been found to affect physical health (e.g., new infections and allergies, but also homelessness); social wellness (e.g., material losses, stress related violence, disruption of daily life) and mental health (e.g., anxiety, depression, PTSD) (Clayton et al., 2014, 2017; Mullins and White, 2019; Généreux et al., 2020).

The consequences of exposure to extreme weather events may also be delayed and even passed on to later generations (Cianconi et al., 2020). Unfortunately, however, it was found that a lack of understanding of climate change and its health implications can lead to inaction, such as clinging to habits that amplify this phenomenon (Koh, 2016). This is of increasing concern as younger people are expressing more and more signs of eco-anxiety as well as discontentment with political inaction (Hickman et al., 2021).

The consequences of climate change also have been found to exacerbate socioeconomic inequalities between and within countries (PNUD [UNDP], 2020; IPCC, 2022) with the most vulnerable being the most affected (Hallegatte et al., 2016; Winsemius et al., 2018). In sum, climate change is a complex problem affecting multiple sectors of human activity (economy, policy, health), and while some research has found that people seek and find comfort and wellbeing in nature (Zelenski and Nisbet, 2014), it has also now become a great source of anxiety for many.

## A paradoxical relationship with nature

As Passmore and Howell (2014) have noted, experiences of close contact with nature may bring about ambivalent emotional states: “intense feelings of awe at the power of nature” (p. 380) can at once make us feel “completely connected to, and at one with, the vastness of the universe [while also] concurrently experiencing a sense of diminished self within this vastness, an experience that can be more terrifying than comforting” (p. 380).

On the one hand, a great deal of research demonstrates the psychological and physiological benefits of regular exposure to nature (Van Gordon et al., 2018). Research on the benefits of natural contact has focused on its calming effects and cognitive benefits (Chalquist, 2009), as well as other positive impacts: on altruistic behaviors (Weinstein et al., 2009), vitality (Ryan et al., 2010), wellbeing, and mental health (Zelenski and Nisbet, 2014; White et al., 2019), on emotional responses, beliefs, attitudes, and pro-environmental behaviors (Kaplan, 1995; Nisbet et al., 2009; Zelenski and Nisbet, 2014; Zelenski and Desrochers, 2021).

On the other hand, individuals and communities are negatively affected by both the direct experience of local natural disasters linked to climate change and regular exposure to news reports dealing with these changes and their often catastrophic effects (Leiserowitz et al., 2013; Reser et al., 2014), which can turn nature into a source of considerable stress (Clayton et al., 2017), or induce eco-anxiety (Pihkala, 2020; Coffey et al., 2021). The latter evokes a chronic state of worry related to helplessly and frustratingly witnessing the irrevocable impacts of climate change upon the immediate future and that of our children (Clayton et al., 2017). Such emotional states are particularly concerning for more vulnerable groups (i.e., children, youth, indigenous people, and remote communities) (Coffey et al., 2021). There is growing evidence that young people (16–25 years old) are overwhelmingly concerned and distressed by the effects of climate change (Hickman et al., 2021).

For the complex and interconnected Earth ecosystem processes, which humanity has been wantonly tampering with, we are now given a life altering paradox (Rockström et al., 2009b) to consider: how far can we go about mastering and submitting the Earth before we ourselves become slaves to the consequences of our own actions? This conundrum can result in ambivalent feelings towards nature: *it can be at once anxiety-provoking as well as a source of peaceful “refreshment” or “rejuvenation.*”

Short of properly recognizing the interdependent nature of the biosphere illustrated by this paradox, how can we then foster wellbeing (i.e., optimal quality of life) for all people living on the planet? How can we be open-minded and celebrate our life-giving relationship with nature while being alert and cautious in our actions toward it at the same time? Should we not explore the deeper meaning of our relationship to nature in light of the magnitude of the task at hand? Paradox is often spirituality’s native tongue (Tickerhoof, 2002; Palmer, 2008); its expression can therefore help us work toward some integration.

## Spirituality

In today’s western societies, spirituality is often presumed to be innate. It is personalized, democratized, and eclectic, drawing from various belief systems and disciplines of thought (Sheldrake, 2011). Given the subjective nature of spirituality, academia has struggled to make sense of this emotionally loaded notion that carries complexity, fluidity, and depth (and mystery): “Spirituality is like the wind – although it can be experienced, observed, and described, it cannot be ‘captured”’ (Nye, 1999, p. 58). The nature or the essence of the spiritual remains immaterial (Pargament, 2007). However, in the field of psychology of religion and spirituality, despite the constant evolution of its definition over time, spirituality is commonly defined as the search for, or communion with, the sacred (Pargament, 2007). Within the PP framework, spirituality is often seen as a character strength; it is understood here as a capability that shapes behavior and provides comfort (Peterson and Seligman, 2004). It refers to having coherent beliefs that promote personal growth, enabling one to understand the universe and one’s place in it in order to contribute to it (Peterson and Seligman, 2004).

Relevant to the context at hand, the study of spirituality in psychology has also two important spiritual dimensions, namely, meaning and connection (and interdependence) (Bellehumeur, 2021). When dealing with climate change, these two specific spiritual dimensions are particularly important since they involve a connection with nature, Superior Being (God), or one’s surroundings that are associated with meaning and quality of life (Paul Victor and Treschuk, 2020). Combining these threads involves finding meaning in protecting humanity’s connection with nature for a sacred purpose that transcends one’s egoistic desires.

## Overview of the three waves of positive psychology in the Anthropocene era

The landscape of PP is also evolving. According to Lomas et al. (2020), there are at least three waves that emerge from this ocean of ideas.

The *first wave of PP* can be attributed to Seligman, in the late ‘90s, who proposed PP as a means of focusing on the positive aspects of people’s lives to foster wellbeing and promote mental health (Seligman, 1998; Seligman and Csikszentmihalyi, 2000; Lomas et al., 2020). With regards to nature and climate change, research has found positive links between (regular contact with) nature and wellbeing (White et al., 2019), mental health (Kuo, 2015), and with enhanced mindfulness practice (Van Gordon et al., 2018), as well as positive links between character strengths and sustainable behavior (Corral-Verdugo et al., 2015). However, criticisms of the first wave of PP included an overemphasis on the positive to the detriment of addressing real and felt difficulties and deficits (Held, 2004), which gave rise to another wave.

The *Second wave of positive psychology (PP 2.0)* was proposed by Wong (2011), and consists of two pillars–existential positive psychology and indigenous psychology–offered as a complement to the limitations of first wave PP. Within PP 2.0, positivity, the key concept of first wave PP, is replaced by polarity, based on the dialectic nature of psychological states (mostly emotions). Embracing the darker as well as the lighter sides of life, as proposed by PP 2.0, is clearly relevant to the “dark” topic of climate change, as one can find it hard to think positively in the midst of a crisis or set aside the stress and losses experienced upon dealing with natural disasters that affect many people at once while being caught in the grip of worsening solastalgia (or eco-anxiety; Pihkala, 2018). PP 2.0 thrives to foster sustainable wellbeing by accepting and courageously confronting the darker sides of life–such as the suffering caused by climate change–while aiming to achieve an adaptive balance through dialectics (Wong, 2019). Yet, another wave is currently emerging.

The *Third wave of PP* (Lomas et al., 2020) involves a new key concept of “greater complexity,” as the PP movement is becoming more interdisciplinary and multicultural, embracing a broader range of methodologies. Going beyond the individual person, it critically examines groups, organizations, and broader systems (e.g., political, economical) in which people are embedded (Lomas et al., 2020). This third wave entails epistemological broadening that is more inclusive and open to ideas beyond the scope of Western positivistic paradigms, making way for “non-positivistic PP” (Lomas et al., 2020).

Focusing mainly on positivity, the first wave of PP uses the Values in Action (VIA) model, which is based on six core universal virtues expressed through 24 character strengths. One of the latter is spirituality, linked to the virtue of transcendence (Peterson and Seligman, 2004). However, spirituality is not seen here as an all encompassing dimension of one’s life. Nonetheless, proposing a VIA model integrating spirituality, Niemiec et al. (2020) have suggested possible reciprocal relationships between spirituality and other character strengths through what they called the grounding path and the sanctification path, both of which lead to greater wholeness. Littman-Ovadia and David (2020) propose a bilateral relationship between character strengths and spirituality (associated with Non-duality).

Second wave PP further integrated the spiritual dimension in line with both Frankl’s existential psychology and the dialectical nature of human experience at the heart of Taoism (Wong, 2011). This Second Wave being more open to spirituality views it as capable of bringing out both the best and the worst in human nature. While the first and second waves of PP mainly focus on individuals, the third wave broadens its breadth of application to groups, communities and systems (Lomas et al., 2020). By integrating qualitative methodologies and epistemologies (including hermeneutics, constructionism, phenomenology, etc.) (Lomas et al., 2020), it may help pave the way for the study of more subjective but meaningful phenomena such as themes pertaining to spirituality (Pargament et al., 2013). Wissing (2022) suggests that this new wave may actually be an early sign of a new domain of inter- or transdisciplinary wellbeing studies. This is better suited to understanding the complex nature of bio-psycho-social-ecological wellbeing, and thus to promoting health and wellness in times of enormous changes and challenges, such as the climate crisis. Table 1 summarizes some of the main epistemological considerations concerning PP and spirituality (Wong, 2011; Lomas et al., 2020).

**TABLE 1:** Epistemological considerations concerning the three waves of positive psychology (PP) and spirituality.

	PP (First wave)	PP 2.0	PP 3.0	Spirituality
Scope/emphasis	Positivity	Polarity	Complexity	Wholeness
Vision of the human person	Compartmentalized	Dialectics; holistic	Holistic, multidimensional	Integrating view
Paradigm	Scientific; disciplinary perspective	Pluralistic perspective (open to existential meaning)	Scientific, expansion in scope (Contextual, Systems informed, Cultural and linguistic; Ethical)	Multiple conceptual and disciplinary frameworks; Integrative perspective
Research methods	Mostly quantitative (self-reported scales) and lab studies	Quantitative, open to qualitative approaches	Quantitative and qualitative (implicit and computational)	Variety of methods (non-disciplinary)
Focus	Mostly the human person (individuals)	Mostly the human person (yet more open to groups)	Beyond the human person (groups and systems)	All people living on Earth (from the past, present and future)

## Concluding remarks and implications

Both PP and spirituality explore common themes (i.e., compassion, growth, hope, forgiveness, virtues, meaning, etc.) pertaining to improving people’s lives and flourishing (Pargament et al., 2013; Shankland, 2019). Since “complexity” means (from the Latin word “complexus”) “weave together” (Morin, 1999, 2000), it invites spirituality to be woven into the PP framework. For instance, linked to a lack of holistic authenticity (Held, 2002), the “tyranny of positivity” has fueled a cultural discourse in which negative emotional states are not only considered undesirable, but also pathological (Lomas and Ivtzan, 2016). Such does not correspond to the human being’s quest for meaning and wholeness (Malette, 2019), which speaks of “cohering harmony” (Wissing, 2022). This calls for more conceptual strands of wellbeing to be woven into the fabric of multidimensional flourishing.

**FIGURE 1:**
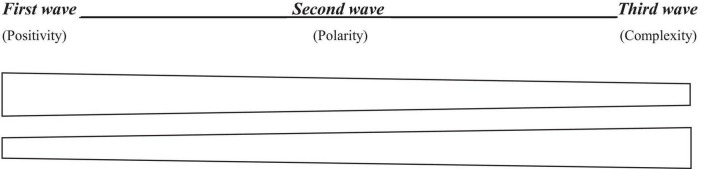
Gradual integration of spirituality in the evolution of positive psychology (PP).

PP 3.0 welcomes non-Western contributions to PP wisdom. And as one characteristic of Western culture (where contemporary psychology was largely developed) within the past century has been secularization (Taylor, 2007), this implies that non-Western contributions will inherently involve non-secular (i.e., spiritual) assumptions. Borrowing from the metaphor of waves (Lomas et al., 2020), it seems that the field of PP has gradually become more open to integrating spirituality since the beginning of the first wave. As shown in Figure 1: there is a move from a compartmentalized view of the first wave of PP (i.e., VIA model) toward a more holistic view of PP 2.0 (i.e., embracing dualistic paradoxes) and onward to even more complexity within PP 3.0 (i.e., multiple systems with multidimensional integration). In Figure 1, the upper band refers to the place (i.e., relative proportion) occupied by positivity within the three waves of PP; the lower band refers to the relative and growing importance of spirituality within these three waves.

Lomas et al. (2020) describe the evolution of PP using the metaphor of “waves” as if born from an ocean of ideas (culture as a whole):

“(…) these energy pulses (i.e., waves) constitute ideas, animating the water and coalescing into visible rolling movement. Thus, people do not ‘belong’ to any particular wave, but rather may be energized by, and moved to contribute to, the passing waves. (…) beneath these waves are deeper forms of movement operating over longer time frames, lasting centuries and even millennia.” (pp. 1–2).

The energy in this metaphor can complement the Chinese proverb: “*The waves do not rise if there is no wind*.” This is because the etymology of the word “spiritual” is related to the word “breath” (i.e., of God) (or *ruah* in Hebrew, *pneuma* in Greek, *spiritus* in Latin, *qì* in Chinese, or *prānā* in Sanskrit) (Lomas, 2019), which is connected to the notions “to breathe,” “breath of life,” or soul. In a sense, when the evolving winds of the soul and a new breath of life blow on the dynamic philosophical assumptions, waves of momentous energy shift. It seems that there are signs that the evolving trends of PP 3.0 and spirituality are beginning to blow in the air, and the conundrums of climate change can receive positive impacts if there is openness.

In terms of implications for integrating spirituality within (third wave) PP, this requires that the notion of meaning be explored in dealing with climate change, adding another dimension. For example, there is the dimension of meaning in protecting the physical environment and wildlife from deterioration. This is distinct from the dimension of meaning in forming friendships and connection with valued animals. Then there are the dimensions of ensuring a sustainable future for one’s grandchildren, protecting one’s conscience as well as the conscience of society, and building a network of allies with other like-minded individuals who can collaborate with collective purposeful energy (i.e., synergy).

All of these subcomponents of multidimensional meaning have the common characteristic of living for a sacred purpose greater than oneself and arguably fits on a scale of purpose that transcends one’s own egoistic goals. At the same time, they have tangible, concrete dimensions to them that can literally be hands on at times. This allows one to simultaneously experience an integration of abstract transcendence and concrete “earthiness” at the same time in multiple ways, which can align congruence with the mind, body, and soul. For instance, choosing to spend time near green and blue spaces on a regular basis, while practicing a mindful presence, can act as a protective factor against stress and eco-anxiety (Van Gordon et al., 2018). Indeed, cultivating mindfulness, seen here as a spiritual practice (Nhat Hanh, 2013), has the potential to bring our sense of connection to the Earth and our compassion to life, generating energy that can transform our intentions into pro-environmental actions.

In the context of confronting climate change, integrating spirituality to the PP framework may encourage the mind to conceptualize (e.g., strategize for environmental sustainability), the body to act (e.g., activity that protects the biosphere), and the soul to contemplate, reflect, and connect with Mother Nature and other like-minded individuals. This prevents a person from being narrowly “tunnel-visioned” in one isolated dimension of dealing with climate change and instead gives a kaleidoscope of a vision of what a multidimensional lifestyle in dealing with climate change can look like for one’s wellbeing.

Returning to the paradoxical relationship between humans and nature, these points further justify integrating spirituality within the field of PP. Echoing the dialectical nature of the psyche, as identified by PP 2.0, we need to fully recognize the existence of paradox so we can find ways to transcend it (Tickerhoof, 2002). This process of transformation may solicit higher levels of consciousness that transcend binary understandings of balance, such as non-dual reality (Davis, 2011; Littman-Ovadia and David, 2020).

Lastly, we merely suggest that the ecospirituality movement, which has been around for decades and is today growing in popularity (Choné, 2017), may serve as a source of inspiration for researchers and clinicians seeking to integrate spirituality to PP in the context of climate change. Ecospirituality gathers people who are concerned with the current ecological crisis and view it as essentially a spiritual crisis of values (Choné, 2017). This ecocentric view recognizes the intrinsic value in all human and non-human forms of life; it suggests to become one with the Earth and reorienting our efforts toward protecting our planet (Egger, 2018). The development of ecological virtues such as sobriety, gratitude and hope are encouraged [i.e., Nhat Hanh (2013)’s ecobouddhism].

In sum, there is an emerging new “consciousness” in which people define their quality of life in spiritual and relational terms rather than materialistic ones (Speth, 2008, 2012). In times of climate crisis, there is a call to respond to the incoming waves of physical, psychological, and philosophical change that demand adaptation and holistic versatility. We can either ignore the incoming wave or ride its momentum toward one’s plenitude or wholeness.

## Data availability statement

The original contributions presented in this study are included in the article/supplementary material, further inquiries can be directed to the corresponding author.

## Author contributions

CRB was the main contributor of this manuscript. CB and CK had made a valuable contribution to the work and approved it for publication. All authors contributed to the article and approved the submitted version.
